# Adopting Telemedicine for the Self-Management of Hypertension: Systematic Review

**DOI:** 10.2196/medinform.6603

**Published:** 2017-10-24

**Authors:** Michael Mileski, Clemens Scott Kruse, Justin Catalani, Tara Haderer

**Affiliations:** ^1^ School of Health Administration Texas State University San Marcos, TX United States

**Keywords:** hypertension, telemedicine, eHealth, mHealth, disease management

## Abstract

**Background:**

Hypertension is a chronic condition that affects adults of all ages. In the United States, 1 in 3 adults has hypertension, and about half of the hypertensive population is adequately controlled. This costs the nation US $46 billion each year in health care services and medications required for treatment and missed workdays. Finding easier ways of managing this condition is key to successful treatment.

**Objective:**

A solution to reduce visits to physicians for chronic conditions is to utilize telemedicine. Research is limited on the effects of utilizing telemedicine in health care facilities. There are potential benefits for implementing telemedicine programs with patients dealing with chronic conditions. The purpose of this review was to weigh the facilitators against the barriers for implementing telemedicine.

**Methods:**

Searches were methodically conducted in the Cumulative Index to Nursing and Allied Health Literature Complete (CINAHL Complete) via Elton B Stephens Company (EBSCO) and PubMed (which queries MEDLINE) to collect information about self-management of hypertension through the use of telemedicine.

**Results:**

Results identify facilitators and barriers corresponding to the implementation of self-management of hypertension using telemedicine. The most common facilitators include increased access, increase in health and quality, patient knowledge and involvement, technology growth with remote monitoring, cost-effectiveness, and increased convenience/ease. The most prevalent barriers include lack of evidence, self-management difficult to maintain, no long-term results/more areas to address, and long-term added workload commitment.

**Conclusions:**

This review guides health care professionals in incorporating new practices and identifying the best methods to introduce telemedicine into their practices. Understanding the facilitators and barriers to implementation is important, as is understanding how these factors will impact a successful implementation of telemedicine in the area of self-management of hypertension.

## Introduction

### Incentives

The Patient Protection and Affordable Care Act of 2010 (PPACA) has put a burden on health care facilities, forcing them to cut costs and focus on quality. We must find effective methods to keep patients with chronic diseases such as hypertension and diabetes from having to come to the facility directly, by allowing them the choice of being monitored from home. This is easily and effectively done through telemedicine, such as telephone-based medicine, electronic medicine, or videoconferencing [1]. Telemedicine has incentives for health care facilities and the patients who would participate. These incentives include increased access to rural areas, increased involvement of nurses, decreased involvement of doctors in menial tasks, potential cost-effectiveness, interactive behavior change, high patient satisfaction, and positive long-term health outcomes [1-5]. Although there are clear advantages and evidence showing that telemedicine improves outcomes of hypertension and other chronic illness not only in the short term but also in the long term, studies show that health care facilities and medical staff are skeptical regarding the adoption of telemedicine models because of the shift of responsibility to the provider to check up on the patient [6].

Incentives for cost cutting and improved outcomes exist through PPACA and Medicare reimbursement policies. By increasing the quality and patient satisfaction, higher reimbursement can be realized through federally funded programs. Telemedicine can be rapidly implemented and can easily negate some of the financial burdens in facilities throughout the United States by higher patient volumes and by more efficient use of patient-physician care time [1].

### Identification and Definition of Key Terms

According to the World Health Organization, telemedicine is “the delivery of health care services, where distance is a critical factor, by all health care professionals using information and communication technologies for the exchange of valid information for diagnosis, treatment and prevention of diseases and injuries, research and evaluation, and for the continuing education of health care providers, all in the interests of advancing the health of individuals and their communities” [7]. The use of telemedicine has expanded vastly to include a “variety of applications and services using two-way video, email, smartphones, wireless tools and other forms of telecommunications technology” [2]. Telemedicine has the potential to impact the health care industry in profound ways with the constant creation and innovation of technology. Currently, the health care system relies on face-to-face communication to deliver care. Telemedicine offers a method to be utilized in conjunction with face-to-face communication with providers. It is not a separate specialty in the medical field because telemedicine is typically integrated in health care institutions within information technology or the delivery of care [2]. There is no distinction between the terms “telemedicine” and “telehealth,” and they are considered interchangeable to encompass a variety of remote health care options [7]. Patient consultations via videoconferencing, transmission of still images, electronic health (eHealth) including patient portals, remote monitoring of vital signs, continuing medical education, consumer-focused wireless applications, and nursing call centers, among other applications, are all considered part of telemedicine and telehealth [7].

### Hypertension Defined

Hypertension is defined as having abnormally high blood pressure [8]. Known as the “silent killer,” hypertension is listed as one of the most important causes for premature death; it affects 1 billion people worldwide, with two-thirds found in developing countries [9]. These numbers are growing at an alarming rate, with an estimated 1.56 billion adults to be afflicted with hypertension by 2025 [9]. Hypertension is a chronic condition that occurs when blood is pumped through the arteries with excessive force [1]. Hypertension can lead to many health risks, including heart attacks, strokes, kidney failure, or other life-threatening health problems [1]. Causes of hypertension can include kidney fluid and salt balances, blood vessel structure, genetic causes, and environmental causes such as unhealthy lifestyles, obesity, and the use of certain medications [8]. In the United States, 1 of every 3 adults has hypertension and only about half of the population with hypertension has the condition under control [9]. This costs the nation US $46 billion each year in health care services and medications required for treatment and missed workdays [9].

### Physician Dependence and Self-Management

Patients are highly dependent on their physicians for information on their health. Self-management refers to taking responsibility for one’s own behavior and well-being. Implementing self-management in the health care setting is the start to educating patients of their current state of health and conditions affecting them. Educating not only improves the individual’s knowledge of the condition but also allows for early detection of health problems and enables the individual to correctly self-titrate medications and allows for timely interventions [2,8].

### Telemedicine Adoption Among Health Facilities

Facilities that have adopted models of telemedicine have shown better patient outcomes and satisfaction, higher patient volumes, and increased facility space to be used for other purposes. Although recent studies have not shown a direct short-term relationship to cost savings, they recognize that in the long-term, cost-effectiveness will be realized [5,10,11]. With the obesity rate in America at an all-time high, along with hypertension diagnoses on the rise, it is important to recognize the benefits that telemedicine offers to consumers. Some of these are sustainable intervention, long-term blood pressure control, improved patient knowledge and accountability, facilitated communication between the patient and the provider, and long-term cost-effectiveness [3,12]. The benefits of telemedicine are yet to be fully realized through research, and it could easily become a regulation to implement it as a tool, such as electronic health records or the International Classification of Diseases, Tenth Edition (ICD-10). It is in the best interests of health facilities to implement this technologically advanced medical care technique into their operations, allowing for a competitive advantage.

### Telemedicine Impact

Although telemedicine offers numerous benefits, the benefits are not fully recognized at the beginning of implementation. With no governmental incentives existing in place for telemedicine, facilities are skeptical to take the leap into this new care technique. This review highlights the facilitators and barriers to implementing telemedicine techniques into health care facilities to identify the benefits that can be realized if adopted [2-4,6,11,12].

### Rationale

The findings of this review will be useful to health administrators, physicians, nurses, and other stakeholders in facilities that are weighing the potential benefits and barriers of adopting a telemedicine policy into their organization. This review is also useful to patients. With the ability of technology to inform the public, patient awareness on the management of their own health has made them a major stakeholder in health care. By extending these findings to the public, increased awareness of the positive outcomes to the adoption of telemedicine might be the push to implementation that the health care facilities need.

## Methods

### Data Collection Process

Information for this review was collected through the use of two databases: Cumulative Index to Nursing and Allied Health Literature (CINAHL) Complete via Elton B Stephens Company (EBSCOhost) and PubMed (which queries MEDLINE). The search focused on self-management of hypertension through the use of telemedicine. The members of the research team reviewed the papers identified during the search and summarized data relative to this review. During successive independent reviews, members compared and discussed the papers and reasons for their inclusion in the study. Papers were included based on their discussion of facilitators and barriers to the self-management of hypertension via telemedicine. The members of the research team had full agreement on all papers included in this systematic review.

### Sample

**Figure 1:**
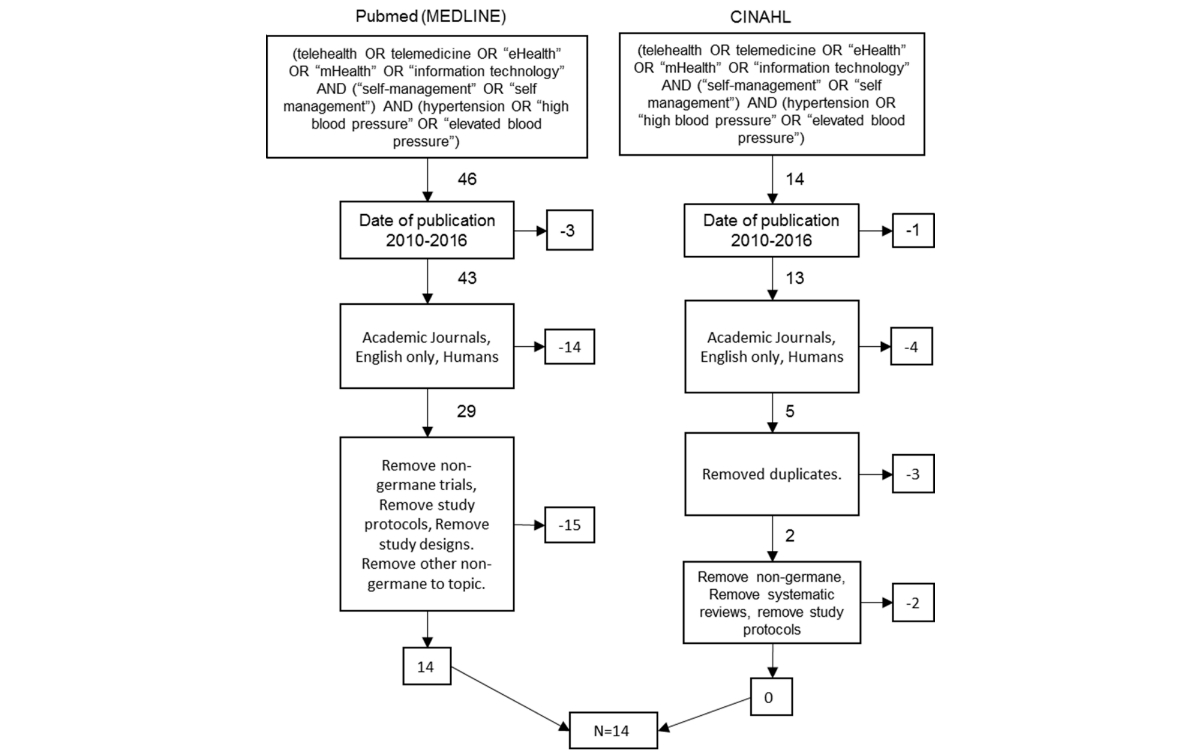
Preferred Reporting Items for Systematic Reviews flow diagram.

Research databases were queried from PubMed and CINAHL using search terms of (“telehealth” OR “Telemedicine” OR “eHealth” OR “mHealth” OR “information technology”) AND (“self-management” OR “self management”) AND (“hypertension” OR “high blood pressure” OR “elevated blood pressure”). Several exclusion criteria were also specified: duplicates were excluded; only academic journals were included; papers were in English only; human-based studies were only included; study protocols and designs were eliminated; and nongermane trials were excluded. Searches were limited to date of publication from 2010 to 2016 (n=14). This process is illustrated in Figure 1.

**Table 1:** Facilitators and barriers associated with the implementation of telehealth in the self-management of hypertension.

Authors	Facilitators	Barriers
McKoy et al [13]	Rapidly evolving technology	Self-management requires much support
	Decreased costs for providing care	Limited oversight, regulation, and guidelines
	Mobile phones can become electrocardiographs (EKG) or other diagnostic machines	Blurring of professional role of practitioners
	Increased access to care that patients otherwise may not receive	Potential for increased liability for practitioners
	Large application to medically underserved areas	Need for identification for ways to mitigate risk
	Ease in scheduling, communication, monitoring, and management for patients and practitioners	
Kumar et al [14]	Apps attached to practitioners have increased oversight of patients and ease of facilitation of care	Many available apps for mobile phones do not have a practitioner involved with them
		Apps available are not documented as valid ways to measure blood pressure
		No oversight to app production or effectiveness
		Lack of Food and Drug Administration approval
Kaambwa et al [3]	Cost-effective in the long run	Differences in results compared with other studies
	Adjusted life years gained	Varying results between men and women
	Markov model	
	Reduced blood pressure compared with usual care	
Maciejewski et al [12]	Sustainable intervention	No economic gains
	Long-term blood pressure control	Not many other studies to exhibit consistency
Wakefield et al [15]	Home-based	No significant difference between intervention and control group
	More timely changes	Information technology unlikely to lead to improved outcomes alone
	Targets remote treatment outcomes	Need for responsive clinical processes
	Effectiveness in the short term	
Shaw et al [16]	Improved patient outcome	Unclear long-term commitment
	Positive organizational culture	Added workload
	Evidence-based and nurse administered	Skeptical staff on positive outcomes
	Information technology infrastructure and support	
	Utilization of existing equipment and space	
Fitzner and Moss [4]	Increased access	Literacy level
	Interactive behavior change technology	Comfort level with technology
	Chronic care model	Security of personal health data
	Technological tools lead to improved patient health	Accuracy of information
	High access to telecommunication	Medicare’s efforts to extend reimbursement to self-management training
	Ease and immediacy of communication	
	Convenience of home	
	Portable	
	Rapid growth of use in mobile phones	
	Effective, efficient, and affordable ways to reach and support minorities	
Melnyk et al [10]	Patient Care Alignment Teams; stresses non-face-to-face interactions	Patients adherence to the guidelines/rules
	Facilitates individualized personal interaction	Self-management components to consider
	Remedies face-to-face intervention problems	
Piette, Marinec et al [5]	Increased access to health information between visits	Interactive voice response call completion rate declined
	Information for users	Technical challenges
	Delivered from long distances	
Jackson et al [17]	Medical and behavioral aspects addressed	
Piette, Datwani et al [18]	Access	Labor intensive and rarely available in low- and middle-income countries
	Cloud computing can make mobile health (mHealth) services more accessible	Lacks the resources to launch and maintain an mHealth service
		Relatively little collaborative work with patients’ clinical teams
		Effort to educate providers
Wang et al [11]	Hypertension is a common reason for men to go to the doctor	Substantial time costs accumulated for nurses to prepare
	Different telephone interventions added with usual care	No long-term difference in results compared with usual care
	Costs may not significantly differ from that of usual care	Hypertension Intervention Nurse Telemedicine Study (HINTS) intervention was costly and time-consuming to deliver
		Unknown whether intervention generates other patient-centered outcomes or efficiencies in other aspects of medical care
Jones et al [6]	Participants valued additional information	Self-management hard to be maintained by participants
	Home blood pressure readings more natural	Borderline readings
	Greater control and more involvement	Self-titration
	Improvement of knowledge for the patient	Needs significant input from general practitioner

### Data Analysis

Narrative summaries related to factors that influenced the adoption of telemedicine for the self-management of hypertension were extracted from each paper and included in Table 1. These, in turn, were grouped into larger recurring themes: either key determinants or impediments to success. The themes chosen were by consensus of the authors. Those chosen were agreed upon to be ones that provided overarching summary to the facilitators and barriers extracted. These themes were then divided into two affinity matrix tables for facilitators and barriers. Each table documents the themes, their citation occurrence, their frequency sum, and their frequency percentage.

## Results

The findings were summarized into the facilitators and barriers table after the members of the research team chose papers to construct the systematic literature review. All identical papers were analyzed and unified before the table was generated. The members of the research team reevaluated the papers and determined facilitators and barriers in the self-management of hypertension via telemedicine. Results are summarized in Table 1. Papers are listed in the order of publication, with the most recent papers at the top.

During the course of the period chosen (2010-2016) for this systematic review, 112 authors published 14 works that specifically studied, analyzed, and discussed factors relating to the self-management of hypertension via telemedicine. Most of these works highlighted both facilitators and barriers, and only one highlighted only facilitators. Papers originated from multiple countries, and a total of 48 facilitators (55%) and 40 barriers (45%) were observed.

## Discussion

### Facilitators

About 17% more facilitators to implementation were noted than barriers (48:40). The authors of this review compared and grouped the facilitators and barriers into common themes. A total of 24 themes were noted between the two categories. A total of 13 facilitator theme categories and 11 barrier categories were identified. Table 2 illustrates and rank orders the themes from the facilitators based on their frequency of occurrence in the literature.

**Table 2:** Facilitating themes associated with the implementation of telemedicine in the self-management of hypertension.

Facilitators	Occurrences	Frequency (N=48) n (%)
Increased access	[4]^a^, [5]^a^, [13]^a^, [18]	7 (15)
Increase in health and quality	[3], [4], [12], [14], [15]^a^, [16]	7 (15)
Patient knowledge and involvement	[4], [5], [6]^a^, [17]	6 (13)
Technology growth with remote monitoring	[4]^a^, [13]^a^, [16], [18]	6 (13)
Cost-effectiveness	[3]^a^, [4], [11], [13]	5 (10)
Increased convenience and ease	[4]^a^, [13], [15]	4 (8)
Facilitates communication	[10]^a^, [16]	3 (6)
Natural readings	[3], [6], [12]	3 (6)
Personalized care	[10], [11]^a^	3 (6)
Utilizing nurses	[16]	1 (2)
Portable	[4]	1 (2)
Timely	[15]	1 (2)
Utilizing space	[16]	1 (2)

^a^Denotes multiple occurrences in the same paper.

Facilitators mentioned most often in the literature were increased access [4,5,13,18] (multiple occurrences per article: 6,12,14) and increase in health and quality [3,4,12,14-16] (multiple occurrences per article: 10). These themes were identified 7 times each, out of 41 total occurrences (17% each). Regarding increased access, authors have noted facilitators where patients found themselves with access to care that they might otherwise not receive [13] and that technology could easily be adapted to use for medically underserved populations [13]. Furthermore, there was an overall increased access to care and telecommunication by the use of telemedicine [4]. The use of the technology also allowed for care to be delivered over long distances, which increased not only access to care but also access to health information [5,18].

Increases in health and quality facilitators were noted in areas that allowed practitioners to have increased oversight of their patients and an easing of the facilitation of care [14,16]. The telemedicine intervention was also noted to be sustainable [12] and could target treatment outcomes remotely [15], which allowed for increased effectiveness in treatment, a gain of adjusted life years, and improved patient outcomes [3,15,16].

The next two most identified themes were patient knowledge and involvement [4-6,17] (multiple occurrences per article: 18) and technology growth with remote monitoring [4,13,16,18] (multiple occurrences per article: 6,12). These themes were identified 6 times each, out of 41 total occurrences (15% each). Patient knowledge and involvement themes mostly focused on how patients could benefit from the use of this technology. Behavior changes [4,17] and additional information and greater control and involvement for patients [5,6] were noted. Increased knowledge for the patients empowered them to make better decisions and have more control of their own care [6,17]. There was also a “value-added” component to the use of telemedicine, as there was additional information available regarding the diagnosis to patients, which otherwise might not have been readily available [6].

Technology growth was also an important theme identified; the more this technology is used, the greater the applications that will be identified [13]. Everyday devices such as mobile phones can be utilized as an electrocardiograph (EKG) or other diagnostic machines [13]. Increased use also will lead to increased options in information technology infrastructure and support [16] and increased tools to use to promote health in patients [4]. This technology could also drive advances in mobile phones and cloud computing [4,18].

The cost-effective nature of the use of telemedicine in the treatment of hypertension was identified 5 times [3,4,11,13] (multiple occurrences per article: 8) (12%). Telemedicine has been shown to be effective, efficient, and affordable in reaching its target population [4]. Furthermore, it is shown to have decreased costs overall for providing care [13], despite the cost of the per-unit provision of care being the same [11]. The long-term use of this as an intervention is where most cost savings are seen [3]. Studies in the area of cost-effectiveness have been weak overall; however, it is believed that there has been a gross underestimation in this area, as the cost of technology has decreased over time [13]. Cost of self-management was shown to be higher overall in some cases, but as it was associated with an increase in quality of life for patients with a decrease in cardiovascular events, a net savings was noted [3]. These results are further verified in reduced overall costs for the care of patients with the use of telemedicine technologies, despite their increased front-end cost overall [4,11].

Increased convenience/ease of the use of telemedicine in the treatment of hypertension was identified 4 times [4,13,15] (multiple occurrences per article: 12) (10%). Patients were able to realize an ease in their ability to receive care from the convenience of their own homes [4,15]. This translated into not only easier access for patients and providers [18] but also more immediate communication between patients and providers [4]. Providers found significant benefits in the areas of scheduling ease, communications with patients, disease monitoring, and disease management [13].

Three theme areas were next tied with each other in incidence. Facilitates communication [10,16] (multiple occurrences per article: 13), natural readings [3,6,12], and personalized care [10,11] (multiple occurrences per article: 17) were all identified 3 times (7% each). An advantage to the use of telemedicine identified was that it alleviates issues regarding interventions in the face-to-face method [10]. Furthermore, communication is facilitated, as the use of telemedicine is able to create a positive organizational culture for patients and practitioners alike [16]. Also of note is that blood pressure readings in the home environment are more natural [6] and are not plagued by the stress of the medical office or “white coat syndrome.” These more natural readings, which are facilitated because of the use of telemedicine, show patients to have decreased blood pressure readings versus normal in office care [13], which in turn assists in long-term blood pressure control [12]. This ties in with personalized care, as telemedicine facilitates individualized personalized interactions [10]. It also fosters care for those who might otherwise not go to the physician for treatment [11] and allows for different interventions outside the normal ones, which would come with usual care for hypertension [11].

The last four facilitators were each mentioned once in the identified papers. Utilizing nurses [16], portable [4], timely [15], and utilizing space [16] were all mentioned one time in the thematic review (2% each). Nurses were able to provide more timely care and intervention than would normally happen in the office environment [15,16]. The technology that is required is portable and can be carried with the patients anywhere they go [4], which allows for increased ability to give timely care [15]. An existing practice can additionally expand its reach and patient base, as the implementation of technology will not take more space resources, and it can utilize existing equipment in most cases [16]. This allows for an increased patient load and an increase in the amount of care given in a timely fashion.

### Barriers

The barrier mentioned most often in the literature was lack of evidence [3,4,11,12,14,16] (multiple occurrences per article: 7,8), which was identified 9 times of 40 total incidences (23%). Lack of evidence was an area that showed many possible concerns. Telemedicine lacks oversight in the area of application production or effectiveness [14]. Additionally, it lacks Food and Drug Administration approval [14]. There is evidence to show that there are differences in effectiveness between men and women [3] and that there are other inconsistencies between the programs (and the research surrounding them) [12]. Due to these inconsistencies, staff seem to be skeptical about the use of technology in this fashion [16], as the accuracy of the information provided [4] and the validity of the results are in question [14]. Furthermore, inconsistencies also question the outcomes generated by the apps and the efficiency of the use of telemedicine to monitor hypertension [11].

The next most-cited barrier was self-management difficult to maintain [5,6,10,13] (multiple occurrences per article: 13,18), which was identified 7 times (18%). This barrier surrounds the ability of patients to support themselves using the technology. There is a large amount of support necessary for patients to be able to use and maintain a telemedicine program on their own [13]. There are also issues identified regarding patients adhering to rules and guidelines surrounding the use of the technology [10] and keeping up with these patients from the provider perspective [6]. The issues observed with the use of this platform include what to do with borderline readings [6] and patients self-titrating their treatments [6]. Getting patient buy-in and continual participation is in question [5]. Table 3 illustrates and rank orders the themes from the barriers based on their frequency of occurrence in the literature.

There are questions that remain regarding no long-term results and more areas to address [11,13,15] (multiple occurrences per article: 6,10) (15%). The lack of long-term studies on this topic has shown a difficulty in the fashioning of responsive clinical processes [15] and a lack of data to show comparison with normal management of hypertension [11]. In fact, one study showed no difference between a telemedicine group and a normally managed group [15]. Many practitioner issues with liability and the mitigation of risk have been raised with the use of telemedicine and its use in the self-management of hypertension [13], with potential for information technology issues causing the technology to not be reliable [15].

There is also a perception that the use of telemedicine will result in a long-term added workload commitment [11,16,18] (multiple occurrences per article: 11,16), as it was identified 5 times (12%). There are significant concerns with time to train staff [11] and increased efforts required to train providers on the new technology [11,18]. There are concerns over the perception that the technology is labor intensive and is only available in affluent countries [18]. Significant concerns were raised over added workload to already overburdened or time-strapped staff [10], increased educational efforts being required [18], and unclear time commitments to roll out and keep telemedicine programs implemented [10].

**Table 3:** Barrier themes associated with the implementation of telemedicine in the self-management of hypertension.

Barriers	Occurrences	Frequency (N=40) n (%)
Lack of evidence	[3]^a^, [4], [11], [12], [14]^a^, [16]	9 (23)
Self-management difficult to maintain	[5], [6]^a^, [10]^a^, [13]	7 (18)
No long-term results and more areas to address	[11], [13]^a^, [15]^a^	6 (15)
Long-term added workload commitment	[11], [16]^a^, [18]^a^	5 (13)
Costly	[4], [11], [12]	3 (8)
Technology challenges	[5], [18]^a^	3 (8)
Significant input by general practitioner needed	[6], [13]	2 (5)
Variation with providers and systems	[13], [14]	2 (5)
Low health literacy level	[4]	1 (3)
Lack of comfort with technology	[4]	1 (3)
Security of data	[4]	1 (3)

^a^Denotes multiple occurrences in the same paper.

Another concern among providers is the cost of using such technology [4,11,12], as it was mentioned in 3 papers (8%). Payers for services (such as Medicare) have made little efforts to pay for services provided via telemedicine [4], and this is slowing implementation. Furthermore, practitioners are concerned over the cost of the delivery of such services possibly outstripping reimbursement [11] and that there might simply be no economic gain to providing services in such a fashion as via telemedicine [12]. Initial concerns that the cost for the use of the technology might be wasted also exist, as one study showed similar probabilities for inpatient admission in those who used technology versus those who did not [12]. An additional study also showed no significant gains in hypertension control with the addition of technology [11]. However, both studies [11,12] were specific to the Veterans Administration population, and findings could be specific to this particular population.

Technology challenges are also a significant barrier [5,18] (multiple occurrences per article: 16), as they are mentioned 3 times (8%). Practitioners are concerned that they lack the resources (technical and financial) to maintain an in-house mHealth service [18]. Simple technical challenges [5] and the perception that there will be little collaboration among clinical teams in the use of the technology [18] are also major concerns.

Several other barriers were found in the literature, which show concern for implementation on many levels. Significant input needed by general practitioner [6,13] was shown in two papers. The concerns here were that the implementation of such technology would blur professional roles in the treatment milieu [13] and that a fear of a significantly increased workload via input exists among these same practitioners [6]. A concern with variation with providers and systems [13,14] was also noted in two papers. There is limited oversight, regulation, and guidelines over telemedicine applications [13], and there is a fear that many applications exist with no oversight at all [14], medical or otherwise. The potential exists to put patients in an unsafe condition and/or without medical oversight and attention. Finally, there exists concern over low health literacy levels [4], lack of comfort with technology [4], and security of data [4]. Many patients already have a low health literacy, and adding technology to the mix simply complicates the already difficult to provide care [4]. These same patients have a lack of comfortability with technology [4], and there are concerns regarding the safety of personal health data [4].

### Population

Hypertension is associated with multiple chronic conditions, including but not limited to diabetes and cardiovascular disease, and there is an ever-increasing need to enhance care to deliver to these individuals [4,13,15,17]. Telemedicine broadens access to people unable to reach health services easily [4,18]. Patients’ involvement in health decisions will increase along with access to health information in between visits [6,18]. Remote data collection, monitoring, and cloud computing will allow for care to be delivered from a distance [3,4,14,18]. This allows doctors to attend to other patients’ needs that cannot be addressed outside a health care facility. Using telemedicine to attend to the management of patients’ health will vastly reduce problems occurring in face-to-face care [17].

### Cost

Cost is often an argument against telemedicine use in health facilities; however, studies show that implementation such as a Tailored Case Management for Diabetes and Hypertension (TEACH-DM) system allows for rapid implementation at low cost [1]. Studies showing the cost-effectiveness of telemedicine point that there is a variation in savings within the short-term use of telemedicine and that this variation can be expected to be steady and to increase in cost-effectiveness over a 2-year period [3,4,14,15].

Although no direct government incentives exist for implementing a telemedicine system, the increase in quality and Medicare reimbursement shows steady increases in reimbursement and patient volumes [10,11,15], and this should be ample incentive in itself to make the move toward this technologically advanced system of care. There are difficulties in understanding why telemedicine is not being heavily implemented throughout the United States despite its cost-effectiveness and research showing positive cost savings and patient outcomes in the long run [14]. However, this study can be used by facilities to raise awareness of the cost-saving potential that could result from the implementation of a telemedicine system.

### Perceptions

Users’ perceptions pose a great level of concern for facilities thinking of adopting a telemedicine program. Users need to understand the value in consistently using telemedicine to assist with self-management of hypertension. The benefits will not be evident immediately [13]. Having patients develop a positive perception will make administering a telemedicine program more desirable. Telemedicine has made it more convenient for users to keep track of their health; it allows users to take measurements from home and immediacy of communication [2,4,5,14]. By enabling the users to track and record their health status, they feel that they have an increase in knowledge with regard to understanding their health condition [5,6,13]. This helps to increase patient participation with their own health and modify behaviors that result in an unhealthy lifestyle [6,11].

Rejection of using telemedicine is not solely derived from the users’ perceptions; it is heavily influenced by the organization deciding whether implementation is in its best interest. Cost plays a key role in conducting a cost-benefit analysis. Since there is little proven evidence suggesting success in gaining a financial return in using telemedicine for long-term conditions, organizations hesitate on its adoption [2,11,12]. Costs may pose an issue if organizations lack proper strategic planning initiatives and fail to adequately address the purpose for adopting telemedicine into their practice. The organization needs to align a telemedicine program in accordance with its mission, vision, and values. Upon alignment, there will be an increase in the quality of care provided to the patients, thus resulting in improved health. Telemedicine has been studied to show an improvement in the quality of life and health of patients who participated in the research studies [4,14]. Even with research that supports expanding the use of telemedicine, some clinical trials show no economic gains [12]; this poses a challenge for pushing the expansion of telemedicine in organizations.

### Implementation

Implementation of a telemedicine program relies heavily on the vision being aligned throughout the organization. With cost-effectiveness and rapid implementation models such as TEACH-DM being available, the only barrier is uncertainty. With facilities moving toward implementation of telemedicine models, it is likely for this uncertainty to be demolished [2,9]. Poor planning has been an extreme issue because of the alignment of goals and vision required throughout the organization to be successful in a telemedicine implementation process [13,14]. When a health care facility implements telemedicine models, the benefits realized by the facility will quickly create a domino effect of implementation of telemedicine by other organizations. This is because of the high levels of competition in health care today, requiring facilities to consistently look for ways to reduce cost and increase quality.

### Adopting a Telemedicine system

Practices may realize the benefits of using telemedicine in the self-management of hypertension after conducting a cost-benefit analysis. This will aid organizations in determining whether the venture is worth adoption. Qualifications for organizations to implement telemedicine programs, at a minimum, are alignment with the mission of the organization and the strategic plan. A common theme throughout the facilitators was an increase in patient health and the quality of care provided. This directly aligns with the goals and requirements established by the PPACA. Legislature is placing an increasing demand on health care organizations to increase the quality of care delivered to patients at a lower cost. Executing a telemedicine program may help alleviate the burden of the PPACA demands and may benefit the health of the population currently living with hypertension. Access is noted to increase with the use of telemedicine by reaching a broader population that may not have easy access to health care resources and transportation, and individuals who require more care such as the elderly.

These facilitators need to be kept in mind when determining a route to abide by PPACA guidelines. The long-term effects of increases in the health and quality of services that will facilitate financial benefits in the near future should override the initial start-up costs. All decisions are associated with costs and benefits; research indicates that the effectiveness of adopting telemedicine programs is growing, as time unveils the positive outcomes that result from the establishment of these programs.

### Limitations

This review provides a collection of up-to-date data associated with using telemedicine and will assist organizations in weighing the costs and benefits associated with its adoption. A shortfall of this study is that all the papers focused on serving different populations with telemedicine. All the papers were related to using telemedicine to measure high blood pressure, but it was the target populations that varied. Some evaluated using telemedicine to reach people in developing countries, the poor and underserved in developed countries, individuals with diabetes, individuals who only speak Spanish, and individuals who are African American.

The extraordinarily vast number of uses for telemedicine made research difficult with regard to narrowing down the target population while still having enough data to conduct a systematic review. Telemedicine is relatively new to assist with self-management of chronic conditions, and there is limited research on the subject. Time is required for facilities to gather data information on the effectiveness of using telemedicine for this purpose, and this study was limited to papers published only within the past 5 years.

### Conclusions

Weighing the costs versus the benefits in the creation of a telemedicine program for self-management of hypertension is essential to decide whether it fits the needs of the organization. This review presented a myriad of facilitating factors and barriers through a meta-analysis and systematic review of up-to-date papers from two academic databases. The information presented is helpful in understanding the benefits of telemedicine and its function in an organization.

## Abbreviations

CINAHL: Cumulative Index of Nursing and Allied Health Literature
EBSCO: Elton B Stephens Company
EKG: electrocardiograph
eHealth: electronic health
HINTS: Hypertension Intervention Nurse Telemedicine Study
ICD-10: International Classification of Diseases, Tenth Edition
mHealth: mobile health
PPACA: Patient Protection and Affordable Care Act of 2010
TEACH-DM: Tailored Case Management for Diabetes and Hypertension
